# An internet-based intervention for adjustment disorder (TAO): study protocol for a randomized controlled trial

**DOI:** 10.1186/s12888-018-1751-6

**Published:** 2018-05-31

**Authors:** Iryna Rachyla, Marian Pérez-Ara, Mar Molés, Daniel Campos, Adriana Mira, Cristina Botella, Soledad Quero

**Affiliations:** 1 Universitat Jaume I, Castellón, Spain; 2 Institut Universitari d’Investigació en Ciències de la Salut (IUNICS), University of Balearic Islands, Palma de Mallorca, Spain; 3 Universidad de Zaragoza, Campus Universitario de Teruel, Teruel, Spain; 4 CIBER de Fisiopatología de la Obesidad y Nutrición (CIBEROBN), Santiago, Spain

**Keywords:** Adjustment disorder, Internet-delivered cognitive-behavioral therapy, Randomized control trial, Effectiveness, Acceptance

## Abstract

**Background:**

Adjustment Disorder (AjD) is a common and disabling mental health problem. The lack of research on this disorder has led to the absence of evidence-based interventions for its treatment. Moreover, because the available data indicate that a high percentage of people with mental illness are not treated, it is necessary to develop new ways to provide psychological assistance. The present study describes a Randomized Controlled Trial (RCT) aimed at assessing the effectiveness and acceptance of a linear internet-delivered cognitive-behavioral therapy (ICBT) intervention for AjD.

**Methods:**

A two-armed RCT was designed to compare an intervention group to a waiting list control group. Participants from the intervention group will receive TAO, an internet-based program for AjD composed of seven modules. TAO combines CBT and Positive Psychology strategies in order to provide patients with complete support, reducing their clinical symptoms and enhancing their capacity to overcome everyday adversity. Participants will also receive short weekly telephone support. Participants in the control group will be assessed before and after a seven-week waiting period, and then they will be offered the same intervention. Participants will be randomly assigned to one of the 2 groups. Measurements will be taken at five different moments: baseline, post-intervention, and three follow-up periods (3-, 6- and 12-month). BDI-II and BAI will be used as primary outcome measures. Secondary outcomes will be symptoms of AjD, posttraumatic growth, positive and negative affect, and quality of life.

**Discussion:**

The development of ICBT programs like TAO responds to a need for evidence-based interventions that can reach most of the people who need them, reducing the burden and cost of mental disorders. More specifically, TAO targets AjD and will entail a step forward in the treatment of this prevalent but under-researched disorder. Finally, it should be noted that this is the first RCT focusing on an internet-based intervention for AjD in the Spanish population.

**Trial registration:**

ClinicalTrial.gov: NCT02758418. Trial registration date 2 May 2016.

## Background

Adjustment disorder (AjD) refers to the clinical symptomatology that appears in response to an identifiable stressful event, such as separation or divorce, job loss, diagnosis of a disease, or family conflicts. In order for the diagnosis of AjD to be made, symptoms must begin within 3 months after the stressor and disappear within a period of not more than 6 months once the stressor or its consequences have terminated. Because the presence of an identifiable stressor is the key characteristic of this disorder, in the DSM-5 [1] AjD was classified under the new category of trauma and stress-related disorders. The same change has been proposed for the ICD-11, along with the proposal of a new diagnostic concept [2]. Despite these improvements, the category of AjD is not yet sufficiently clear [3] and a recent review study [4] revealed that there is still little support for the ICD-11 proposal of two symptom structure of AjD (preoccupation with a stressor or its consequences and failure to adapt).

According to the different studies carried out so far, AjD is a very common condition [3, 5, 6]. It is estimated to have an incidence of between 5 and 20% in mental health services, and about 50% in psychiatric consultation settings [1]. AjD is also one of the most frequent diagnoses in patients with organic diseases and surgical interventions [7–11], and in cases of absenteeism and work disability [12, 13]. In addition to being a highly prevalent disorder, AjD causes considerable distress and marked impairment in different functional areas of patients’ lives (e.g., family, friendships, school/work, etc.), and it may increase the risk of suicidal thinking and behavior [14, 15].

Despite these worrisome facts, little research has been conducted to identify and develop evidence-based interventions (EBI) for AjD. To the best of our knowledge, no specific EBI are available for AjD, just some suggestions and recommendations [3, 16]. Furthermore, only a few interventions for AjD have been assessed in a randomized controlled trial (RCT) [13, 17, 18]. Cognitive Behavioral Therapy (CBT) predominates in all of them, although other approaches are also included, such as the use of mindfulness.

In any case, the availability of an effective intervention does not guarantee that it reaches everyone who might need it. Internet-delivered cognitive-behavioral therapy (ICBT) might be a feasible solution for this problem. Currently, data on the efficacy of ICBT are available for a wide range of psychological disorders, including stress-related disorders [19–23]. Some of the main advantages of these kinds of interventions are confidentiality, cost savings, flexibility because patients can access the treatment at any time and from anywhere, and the possibility of reaching patients who would otherwise never receive psychological assistance [24, 25].

Three brief computer-based interventions are available for the treatment of AjD symptoms. “iCanADAPT Early” is a transdiagnostic ICBT designed to treat depression and anxiety disorders in cancer settings [26]. Although the program can be used for the treatment of AjD, it was not developed specifically for this condition. Moreover, the inclusion of cancer-specific CBT skills hinders the use of “iCanADAPT Early” with patients who suffer from AjD due to other stressful events. Seren@ctif is a stress management program based on CBT, developed to treat anxiety related to stress [27]. The program focuses only on AjD with an anxiety subtype, and it is not yet accessible via the Internet. Patients have to go to the hospital and access the program on one of the computers available there. Finally, BADI is an online intervention for AjD that includes CBT, mindfulness, and body-mind practices [28]. The program presents a modular format, giving users the possibility of choosing the content they want to work on. Preliminary positive findings were recently published for a BADI intervention [29]. However, the high dropout rates were identified as the primary limitation of the intervention, and they were attributed to its modular and unguided format.

The only self-help intervention for AjD validated to date is a bibliotherapy manual developed by Bachem and Maercker [30] for burglary victims. It is based on cognitive behavioral techniques that have been validated for the treatment of depressive, anxiety, or post-traumatic stress disorders, including behavioral activation, exposure, cognitive restructuring, and relaxation. The manual has been shown to be a feasible and effective solution for AjD symptoms. However, it has not been validated for AjD resulting from other stressors.

Given the impact and prevalence of AjD, we have developed TAO (*Trastornos Adaptativos Online*). TAO is the first online manualized intervention protocol for AjD developed for the Spanish-speaking population. The linear format of the program makes it possible to progressively start to solve the problematic situation. It is based on the CBT intervention protocol developed by Botella, Baños, and Guillén [31], which, to the best of our knowledge, is the first protocol specifically designed for AjD, showing efficacy in several studies [17, 32]. The aim of this study is to present the RCT that will be conducted to examine the effectiveness of TAO in reducing the distress and clinical symptoms of AjD, compared to a waiting list control group. Additionally, the level of patients’ acceptance and satisfaction with the intervention will be assessed.

## Methods/Design

### Study design

**Fig. 1:**
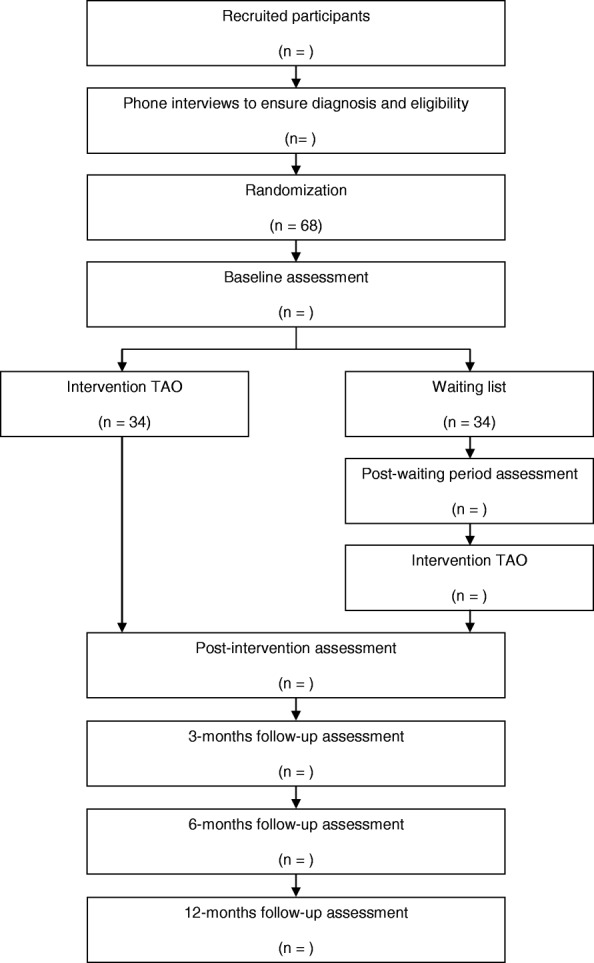
Flowchart of participants

The study is designed as a two-armed, single-blind, parallel group RCT. The trial was registered on the ClinicalTrial.gov database as NCT02758418, and it will be conducted following the Consolidated Standards of Reporting Trials (CONSORT) [33], the CONSORT extension for Electronic and mobile Health Applications and onLine TeleHealth interventions (CONSORT-EHELTH) [34], and the SPIRIT guidelines (Standard Protocol Items: Recommendations for Intervention Trials) [35, 36]. All suitable participants for the trial will be randomly allocated to the intervention group (ICBT) or the Waiting List Control Group (WL). The online informed consent form will be signed before the randomization. Outcome measures will be assessed at baseline, post-intervention, and 3-, 6-, and 12-month follow-ups, in order to provide data on intervention effectiveness and maintenance of the improvements achieved. Fig. 1 displays the flow chart of the study design.

### Recruitment

The trial will be announced on local media (radio, TV, newspaper…) and the website of Universitat Jaume I, and disseminated through social networks (i.e., Facebook and Twitter). The advertisement will be published in newspapers, and information brochures will be placed on noticeboards at local universities (Universitat Jaume I and Universitat de València) and nearby towns.

People interested in the study will be encouraged to send an e-mail to tao@uji.es. The clinical team involved in the study will respond to all e-mails within 24 h and arrange a telephone interview. The interview will last about 40–60 min, and its purpose will be to explain the terms of the clinical trial and check the fulfillment of eligibility criteria. The diagnostic interview will be administered to potential participants during this telephone call.

### Eligibility criteria

In order to be included in the trial, participants must meet the following inclusion criteria: (1) be 18 years old or more; (2) meet DSM-5 [1] criteria for AjD; (3) be able to understand and read Spanish; (4) be able to use a computer and have access to the Internet; (5) have an e-mail address. On the other hand, assessed participants who meet any of the following criteria will be excluded from the trial: (1) receiving another psychological treatment for AjD; (2) meet criteria for another severe mental disorder: alcohol or other substance abuse or dependence, psychotic disorder, dementia, or bipolar disorder; (3) meet criteria for a severe personality disorder or illness; (4) presence of risk of suicide or self-destructive behaviors. Undergoing pharmacological treatment is not an exclusion criterion during the study period, but any increase and/or change in the medication during the study period will imply the participant’s exclusion from subsequent analyses. A decrease in pharmacological treatment is accepted.

The decision about each participant’s inclusion or non-inclusion will be made by the entire clinical team, ensuring a more objective and reliable diagnosis. The telephone interviews will also be recorded, with the patient’s agreement, making independent inter-judge assessment possible.

### Randomization and blinding

Participants included in the study will have to sign the participation agreement without having a priori knowledge about their group assignment. Study researchers will also be blind to the group to which the assessed participants will belong. Once the online informed consent has been signed, an independent researcher will perform a “blocked randomization”, guaranteeing that the same number of participants are allocated to each condition (ICBT or WL). This allocation will be performed following a random number sequence generated by the Epidat 4.1 program.

### Sample size

The sample size for the trial was calculated following the method described by Campbell, Julious, and Altman [37], and Freiman, Chalmers, Smith, and Kuebler [38]. G*Power 3 software [39] was used to facilitate power analysis.

Because there is no published research on the effectiveness of ICBT for the treatment of AjD, the sample size was calculated taking into account outcomes found in trials that used the BAI and BDI-II as measures of clinical change after an ICBT intervention in patients with clinical depression or anxiety disorder [40–42]. After reviewing the literature and adopting a more conservative approach, an effect size of .70 was assumed in the present study. Considering a significance level of 5% and a power of 80%, 26 participants in each group would be enough to detect the assumed difference. However, because the literature reveals dropout rates from ICBTs of around 30% [43], a sample of 68 participants will be recruited (34 per group).

### Ethics

The protocol for this study has been approved by the Ethics Committee of Universitat Jaume I (Castellón, Spain), and the study will be conducted in compliance with the Declaration of Helsinki and good clinical practice. Participation will be completely voluntary. Participants will also be informed that they may leave the study at any time.

The RCT will be carried out in accordance with current EU and Spanish legislation on privacy and data protection. In order to protect the privacy of the participants, all personally identifiable information will be replaced by a randomly assigned username and only made available to the researchers responsible for its supervision. All data from outcome measures and post-module assessments will be stored separately from the personal information and protected according to AES (*Advanced Encryptation Standard*).

### Study groups

#### Adjustment disorders online (TAO)

Adjustment Disorders Online (TAO) is an ICBT based on a manualized protocol for the treatment of AjD, structured in a therapist handbook and a patient handbook. TAO comprises the following therapeutic components: psychoeducation, techniques to manage negative emotions, exposure, problem-solving techniques, mindfulness, acceptance and elaboration of the stressful event, positive psychology strategies, and relapse prevention. It is the optimized version of the original intervention protocol for AjD developed by Botella et al. [31]. More specifically, TAO also includes behavioral activation for mood disturbance, problem-solving techniques to improve the capacity to deal with everyday challenges, and mindfulness to become aware of the thoughts and feelings related to the stressful event instead of trying to escape from them.

The intervention is easily accessible over the internet at https://www.psicologiaytecnologia.com/. In order to provide a more enjoyable experience, the program content is presented through texts, videos, pictures, vignettes, and interactive exercises. Different contents can also be downloaded as PDF files so that users can review them offline.

TAO is organized into seven sequential modules (see Table 1), and it takes about 7 to 10 weeks to complete it. Although users are encouraged to advance one module per week, some modules may require more time. Therefore, the program also emphasizes that everyone should progress at their own pace, dedicating enough time to understand the module contents and carry out the proposed activities.

**Table 1:** TAO content

Module	Aims of the module	Contents
0. Welcome module: starting this program.	- Providing information about TAO. - Promoting the adherence to the program. - Enhancing motivation for change.	- Information about the contents of each module. - Recommendations to get the maximum benefit from the program. - Meditation on reasons to change. - Goal setting.
1. Understanding emotional reactions.	- Providing information about AjD and common reactions to stressful events. - Learning of strategies to manage negative emotions.	- Psychoeducation. - Behavior activation. - Slow breathing technique.
2. Learning to deal with negative emotions.	- Facing avoided situations that contribute to the maintenance of the problem. - Improving the ability to deal with everyday challenges.	- Exposure. - Problem solving technique.
3. Accepting problems.	- Becoming aware of the personal experiences related to the stressful event. - Elaborating and processing the stressful event through the acceptance of the problematic situation.	- Mindfulness. - The Book of Life: Acceptance. - Elaboration of a metaphorical meaning for the stressful event.
4. Learning form problems.	- Starting to see problems as opportunities to grow and learn. - Elaborating and processing the stressful event through the confrontation of the problematic situation. - Promoting personal growth.	- Psychoeducation on the positive contribution of problems. - The Book of Life: Confrontation. - Development of personal strengths.
5. Changing the meaning of problems.	- Elaborating and processing the stressful event through the development of a new meaning for the problematic situation. - Developing a new attitude towards problems.	- Elaboration of a new metaphorical meaning for the stressful event. - The Book of Life: Change the meaning. - Letter of projection towards the future. - Choice of a personal life motto.
6. Relapse prevention.	- Assessing achievements accomplished so far. - Reviewing of leaned techniques. - Identifying problematic situations an developing a plan to deal with them.	- Review of the therapeutic achievements. - Action plan to deal with future problems.

All the modules present the same structure: 1) module agenda; 2) therapeutic contents of the module; 3) exercises and activities to put the psychological techniques learned in the module into practice; 4) assessment of the knowledge acquired during the module; 5) tasks to be completed before advancing to the next module; and 6) summary of the module. An effort was made to simplify the language used in TAO to make it easily understood by users, regardless of their socio-demographic features. Regarding the ease of use of TAO, preliminary results obtained in an acceptance and usability study performed with clinical psychologists and patients with AjD showed that the program interface is highly intuitive and user-friendly and does not require any previous training [44].

#### Waiting list control group

Participants on the waiting list group will be assessed before and after a period of 7 weeks. After completing a post-waiting period assessment, they will be offered the TAO program.

### Support

Because the literature shows that guided ICBT provides better results than completely unguided interventions [45], all the participants will receive weekly phone support. This support will consist of a short phone call (maximum 10 min) during the intervention stage. The aim of these phone calls will be: 1) to clarify doubts about the use and functioning of TAO; 2) to remind them of the importance of continuing to work on the program contents; and 3) to congratulate them for their effort and achievements. Patients will receive up to 10 telephone calls over a 7–10 week period, and so they will have a maximum of 100 min of therapeutic support. No additional clinical content will be provided during the phone calls.

The support will be provided by experienced psychologists who will have at least a Master’s degree in Clinical Psychology. Before taking part in the trial, they will receive training in order to ensure that everyone provides the same support.

### Assessment

Measurements will be taken at five different moments: baseline, post-intervention, and three follow-up periods (3-, 6- and 12-month). The diagnostic interviews will be administered by a trained clinician by phone. Moreover, all interviewers engaged in the assessment of potential participants will be supervised by a clinical team composed of mental health professionals with extensive experience in the diagnosis and treatment of stress-related disorders. Questionnaires will be self-administered online via the same virtual platform as the intervention program. Table 2 provides an overview of the measures used at each time point.

**Table 2:** Study measures and assessment times

Assessment moment	Telephone assessment performed by a therapist	Automatic online assessment
BL	Diagnostic Interview for Adjustment Disorders, ADIS-IV-L*	BDI, Suicide item, BAI, ISL, PTGI, PANAS, MQLI
Post-M	–	Post-module assessment scale, suicide item
Post-M1	–	Post-module assessment scale, suicide item, Expectation of treatment scale
Post-T	Diagnostic Interview for Adjustment Disorders	BDI, Suicide item, BAI, ISL, PTGI, PANAS, MQLI, Opinion of treatment scale
FU	Diagnostic Interview for Adjustment Disorders	BDI, Suicide item, BAI, ISL, PTGI, PANAS, MQLI

*BL,* Baseline; *Post-M,* post-module; *Post-M1,* post-module 1; *Post-T,* post-treatment; *FU*, follow-ups; *ADIS-IV-L,* Anxiety Disorders Interview Schedule for DSM-IV: Lifetime Version; *BDI,* Beck Depression Inventory - Second Edition*; BAI,* Beck Anxiety Inventory; *ISL,* Inventory of Stress and Loss; *PTGI,* Posttraumatic Growth Inventory; *PANAS,* Positive and Negative Affect Scale; *MQLI,* Multidimensional Quality of Life Questionnaire; * used only when differential diagnosis is needed

#### Diagnostic interviews

##### Diagnostic interview for adjustment disorders

This interview will be used for the diagnosis of AjD and to check the fulfillment of inclusion/exclusion criteria. It is a semi-structured interview developed by our research group, taking into consideration the diagnostic criteria for AjD included in the DSM-IV-TR [46], the ICD-10 [47], and the *Structured Clinical Interview for DSM-IV (SCID-CV)* [48]*.* The first part of the interview aims to explore the presence of a stressful event (current or past). In order to make the interview easier, a list of 46 possible stressors is included. The second part includes 28 symptoms related to AjD. The presence and severity of these symptoms is rated on a 9-point scale (0 = Not at all; 8 = Very severe). The validation of this instrument is currently in process.

##### Anxiety disorders interview schedule for DSM-IV

*Lifetime Version (ADIS-IV-L)* [49]. This semi-structured interview will be used only when differential diagnoses with Generalized Anxiety Disorder and/or a Major Depressive Episode are needed. The ADIS-IV-L allows a reliable diagnosis of current and lifetime anxiety, mood, somatoform, and substance use disorders.

#### Primary outcome measures

*Beck Depression Inventory - Second Edition (BDI-II)* [50]*,* validated in the Spanish population [51]. The BDI-II is a widely used self-report inventory that measures characteristic attitudes and symptoms of depression. The total score is obtained by adding the scores on the 21 items that make up the instrument, with a maximum of 63 points. The instrument has good internal consistency (Cronbach’s alpha of 0.76 to 0.95) and test-retest reliability of around 0.8.

*Beck Anxiety Inventory (BAI)* [52], validated in the Spanish population [53]. The BAI measures the severity of both physiological and cognitive symptoms of anxiety. The 21 items are rated on a 4-point Likert-type scale (from 0 to 3), and the total score, which ranges between 0 and 63, is obtained after directly adding together the scores on all the items. Psychometric analyses carried out so far show excellent internal consistency (Cronbach’s alpha ≥0.85).

#### Secondary outcome measures

##### Inventory of stress and loss (ISL)

This inventory is an adaptation of the Complicated Grief Inventory [54]. It consists of 17 first-person statements about the degree to which the lost person/situation interferes in the individual’s life. There are 5 response options, ranging from 0 (“Never”) to 4 (“Always”). The validation of the instrument is currently in process. However, preliminary validation data [55] show excellent Cronbach coefficients in both general (0.91) and clinical AD (0.86) Spanish populations. Test-retest reliability was also excellent (0.90).

##### *Posttraumatic Growth Inventory (PTGI)* [56]

The PTGI is a 21-item instrument that assesses positive outcomes reported by individuals who have experienced traumatic events. A 6-point Likert response format is used, so that each statement is rated from “I did not experience this change as a result of my crisis” (scored 0), to “I experienced this change to a large degree as a result of my crisis” (scored 5). The instrument has excellent internal consistency (Cronbach’s alpha of 0.90) and acceptable test-retest reliability of around 0.71.

##### *Positive and Negative Affect Scale (PANAS)* [57]

The PANAS consists of 20 items that evaluate two independent dimensions: positive affect (PA) and negative affect (NA). The range for each scale (10 items on each) is from 10 to 50. The Spanish version has demonstrated high internal consistency (0.89 to 0.91 for PA and NA, respectively, in women, and 0.87 and 0.89 for PA and NA, respectively, in men) in college students.

##### *Multidimensional Quality of Life Questionnaire (MQLI)* [58]

This is a 10-item self-report instrument that assesses physical and emotional well-being, self-care, occupational and interpersonal functioning, community and services support, personal and spiritual fulfillment, and the overall perception of quality of life. Satisfaction in each of these areas is measured using a 10-point Likert rating scale. The MQLI is brief and easy to administer. It also presents good internal consistency (Cronbach’s alpha of 0.79) and a test-retest reliability index of 0.89.

#### Opinion measures

##### *Expectations and Treatment Opinion Scale* (adapted from Borkovec & Nau [59])

This self-report inventory measures patients’ expectations before they start the treatment and their satisfaction when they complete the treatment. The 6 items are rated from 1 (“Not at all”) to 10 (“Highly”) and provide information about the extent to which: 1) the treatment is perceived as logical; 2) patients are satisfied with the treatment; 3) the treatment would be recommended to a friend with the same problem; 4) the treatment would be useful to treat other psychological problems; 5) patients perceive the treatment as useful for their particular problem; and 6) the treatment is perceived as aversive. Participants will answer the Expectations scale after the therapist explains the rationale for the treatment they will receive and before beginning the treatment. The Satisfaction scale will be completed once the treatment ends. This adaptation has been used in previous studies [60–62].

##### Opinion scale

This 8-item instrument was developed specifically for this trial in order to get more feedback about the participants’ opinions about TAO. Four of the items are answered using an 11-point response scale, rating different statements from 0 (“Not at all”) to 10 (“Very much”): (1) usefulness; (2) attractiveness; (3) convenience; (4) recommendation. Then, four short-answer questions are included to collect qualitative data about: (1) the most useful module; (2) positive features of the intervention; (3) negative features of the intervention; and (4) the overall opinion. This scale will be filled out at post-intervention.

#### Suicidal risk

The presence, frequency, and severity of suicidal thoughts will be assessed during the *Diagnostic Interview for Adjustment Disorders* administered by phone. The inclusion of a suicide item after each program module, at post-intervention, and at follow-up assessments will make it possible to detect participants who are at risk of suicide during the intervention and once the intervention is over.

#### Other post-module measures recorded by the system

The post-module assessment will be performed using a short scale developed by the clinical team involved in the present trial. In addition to suicidal risk, the following variables will also be assessed: the general mood using a 7-point face rating scale, and the intensity of several emotions (joy, sadness, anger, hope, anxiety, relaxation, pride, and guilt) on a 7-point numeric scale. Finally, 10-point numeric scales will explore: (1) the feeling of self-efficacy to deal with the stressful event that caused the AjD; (2) acceptance of negative events; (3) openness to new experiences; and (4) satisfaction with the TAO module.

### Data analyses

The statistical package IBM SPSS Statistics version 22.0 for Windows will be used for data analyses. Baseline differences between groups will be explored for continuous and categorical measures using both t-tests and chi-square tests. Repeated-measures ANOVAs will be used to assess within-group changes over time in primary and secondary outcome measures. Effect sizes will be estimated using Cohen’s d. Linear regression models will be used to study the effect of different variables (e.g., gender, age, and treatment expectations) on adherence and response to the intervention. Any participants who do not complete the post-intervention assessment will be considered drop-outs. On the other hand, the number of times each patient uses the program will be used as the measure of adherence.

Before analyzing the data, a review of state-of-the-art analytic methodology for RCT will be carried out in order to ensure the use of the most suitable statistical analyses. Finally, following SPIRIT and CONSORT guideline recommendations, both intention-to-treat and per-protocol analyses will be reported [34, 36].

## Discussion

According to the evidence, AjD is a common and disabling disorder. The lack of specific treatment guidelines for this disorder often results in the worsening of clinical symptoms because patients do not receive appropriate help. Although different psychological techniques have been found to be useful for its treatment, no EBI are yet available for AjD. In addition, evidence suggests that a large percentage of patients with mental disorders remain untreated, partly due to a lack of personal and primary health care resources, which indicates the need to research and develop new ways to deliver high quality interventions. Therefore, this study protocol describes a RCT to test the effectiveness of an ICBT for AjD (TAO), compared to a waiting list control group.

One of the main strengths of TAO is that it is based on a manualized intervention protocol that has already shown its efficacy in the traditional, face-to face format. The experience with this protocol provided the opportunity to optimize its effectiveness, focusing on active treatment components and adding techniques that clinicians and patients considered important. The TAO’s linear approach allows the progressive acquisition of different skills needed to cope with distress in a gradual but effective way. Because the program was designed to be implemented in patients suffering from mild to severe symptoms, we think the linear structure may be more beneficial than a modular system where patients can freely choose the contents they want to work on. TAO not only provides techniques for the management of distress caused by the stressful event, but it also emphasizes the importance of its reprocessing and gives it a new positive meaning. This reprocessing involves the exposure to thoughts, emotions, memories, and stimuli related to the event, and it can be highly stressful in some cases. Therefore, the use of linear programs like TAO ensures that patients have the resources they need for the successful completion of the task, thus reducing the number of dropouts.

Apart from testing the effectiveness of the web-based intervention for AjD, the RCT will also provide data about TAO’s acceptability to patients and their satisfaction with it. This information will be crucial for the effective implementation of the program in health care settings because the barriers that currently prevent us from taking full advantage of these ICBTs, despite their demonstrated effectiveness, can be broken down.

Finally, personal growth achieved during the intervention period will also be assessed. According to WHO (1948), health is “A state of complete physical, mental and social well-being, and not merely the absence of disease or infirmity”. Huber et al. [63] also emphasize “the ability to adapt and to self-manage, in the face of social, physical and emotional challenges”. Consequently, the reduction in clinical symptomatology might be insufficient to achieve an optimal state of health. Improvements in coping strategies, however, might increase the ability to successfully overcome future challenges, without developing a sense of helplessness and/or AjD. Therefore, the main goal of TAO is to provide strategies to deal with current and future difficult situations, whether or not they can be resolved.

However, the study has limitations. The main limitation is the lack of an active treatment control group for comparison. However, because AjD is considered a transient condition [3], it is useful to explore whether brief interventions like TAO can prevent the chronification of this disorder and the development of more severe symptomatology. Moreover, given the lack of EBI for AjD, the comparison with a waiting list control group could be the first step in the validation of psychological treatments for this condition. Another limitation is that the decision about whether to include participants or not is based on the AjD diagnosis made using the *Diagnostic Interview for Adjustment Disorders*, which is not a validated instrument. The use of other diagnostic tools, such as the Adjustment disorder new model questionnaire (ADNM-20) [64], would have helped to corroborate the diagnosis. Unfortunately, there are no diagnostic instruments for AjD adapted and validated in Spanish for the beginning of RCT.

On the other hand, one potential difficulty in implementing the study might be the dropout rates. According to the literature, around 30% of those who start an ICBT do not complete the program. Preliminary results on the engagement in the BADI modular intervention for AjD showed a dropout rate of more than 80% in the intervention condition [29]. However, the inclusion of telephone support in the present study and the linear format of the TAO might improve the engagement of the patients who use it.

In sum, despite the limitations, the study represents an important attempt to improve access to an EBI that targets one of the most prevalent mental health problems. Showing the effectiveness of TAO might facilitate the inclusion of ICBT interventions within the National Health System, reducing the current waiting lists and improving the quality of the psychological care provided. As Kazdin [65] points out, technology-based interventions like TAO are designed to extend the reach of EBIs and, thus, reduce the burden of mental disorders.

## Abbreviations

ADIS-IV-L: Anxiety Disorders Interview Schedule for DSM-IV Lifetime version
AES: Advanced Encryptation Standard
AjD: Adjustment disorder
BAI: Beck Anxiety Inventory
BDI-II: Beck Depression Inventory, Second Edition
CBT: Cognitive Behavioral Therapy
CONSORT: Consolidated Standards of Reporting Trials
CONSORT-EHELTH: CONSORT extension for Electronic and mobile Health Applications and onLine TeleHealth interventions
DSM-5: Diagnostic and Statistical Manual of mental disorders, 5th edition
DSM-IV-TR: Diagnostic and Statistical Manual of mental disorders, 4th edition - text revision
EBI: Evidence Based Interventions
EU: European Union
ICBT: Internet-delivered Cognitive-Behavioral Therapy
ICD-10: International Classification of Diseases, 10th revision
ICD-11: International Classification of Diseases, 11th revision
ISL: Inventory of Stress and Loss
MQLI: Multidimensional Quality of Life Questionnaire
PANAS: Positive and Negative Affect Scale
PTGI: Posttraumatic Growth Inventory
RCT: Randomized Controlled Trial
SCID-CV: Structured Clinical Interview for DSM-IV, Clinician Version
SPIRIT: Standard Protocol Items - Recommendations for Intervention Trials
TAO: Adjustment Disorders Online, web-based intervention for Adjustment Disorders
WHO: World Health Organization
WL: Waiting List
